# Development of sorghum‐based shortbread biscuits from “muskwari” flour

**DOI:** 10.1002/fsn3.1574

**Published:** 2020-05-29

**Authors:** Roger Djoulde Darman, Matsowa Bouopda Sidoine, Venassius Wirnkar Lendzemo

**Affiliations:** ^1^ National Advanced School of Engineering of Maroua University of Maroua Maroua Cameroon; ^2^ The Higher Institute of The Sahel University of Maroua Maroua Cameroon; ^3^ Ekona Research Center Institute of Agricultural Research for Development Cameroon Bamenda Cameroon

**Keywords:** flour, Muskwari, shortbread biscuits, sorghum

## Abstract

In order to produce biscuits from off‐season sorghum, a local “Muskwari” sorghum was milled and sieved. This flour was used to produce shortbread biscuits with different substitutions rates of wheat flour to that of sorghum. The standard formulation of this same type of shortbread biscuits was used and biscuits were produced with incorporation rates of wheat flour to that of sorghum, from 0% to 100%, with a gap of 10 between two consecutives percentages. The technological characterization of the sorghum flour produced indicates a good water absorption capacity, and interesting solubility index and swelling rate. Technological aspect indicated that by changing speed and kneading time, resting the dough, it is possible to produce 100% sorghum flour shortbread biscuits. Shortbread biscuits made from 70% of wheat flour incorporation had the best average scores for overall preference criteria (6.97 ± 1.30), color (7.1 ± 1.45), and texture (6.62 ± 1.54). For smell and taste criteria, the 40% biscuits and the witness received the highest average scores, respectively, namely 6.77 ± 1.55 for smell and 7.12 ± 1.29 for taste. Analysis of the nutritional and energy intake of the control biscuit and the 70% substitution revealed that between the two, the latter had a significantly higher intake of total carbohydrates (58.51 g), dietary fiber (2.15 g), and total energy (454.1 kcal

## Graphical abstract

In order to produce shortbread biscuits from off‐season sorghum, a local “Muskwari” sorghum was milled and sieved. The obtained flour was of 0.5 mm grain size. This flour was used to produce shortbread biscuits with different substitutions rates of wheat flour to that of sorghum.

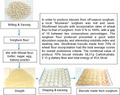

## 1 INTRODUCTION

The Far North region of Cameroon is the first nationwide in cereal production (Guei, Barra, & Silue, 2011). This is due to its high production of sorghum, which averages 778,112 tons, 26.92% of national grain production (Danbe et al., 2018; Weltzien, Rattunde, Mourik, & Ajeigbe, 2018). Despite this, the use of this cereal lack diversification (Anglani, 1998; Miafo, Koubala, Kansci, Saha, & Fokou, 2016; Vunyingah & Kaya, 2016). In fact, 60%–80% of the sorghum produced is destined for self‐consumption (Nienie, Foudjet, & Mallo, 2017; Ronald & Roger, 2017) in the form of ready meals such as couscous, porridge, and local sorghum beer (bili bili) (Favier, 1977; Kengap, Djou, Tchamda, Martin, & Bricas, 2016). Of all these forms in which sorghum is used in Cameroon, there appears to be no transformation on a semi‐industrial and/or industrial scale into products such as biscuit, pastry, and bakery products, although potential market for these products continues to grow in the country (INS, 2015) and in the northern part in particular. Indeed, the urban dynamics observed in the region favors changes in food and culinary habits: we see the emergence of more needs in terms of light, rich foods (bread, biscuits, porridge, donuts, beers, and compound flours that can combine sorghum and wheat, gritz, parboiled sorghum, puffed products, etc.) (Sangwan & Dahiya, 2013; Sathya, 2018). This represents an undeniable opportunity to diversify the use and therefore valorization of local cereals like sorghum, which is the identity of the area. The objective of this study was to produce shortbread biscuits based on “Muskwari” sorghum flour to valorize this cereal for industrial use in the area.

## 2 MATERIALS AND METHODS

### 2.1 Biological material and ingredients

The plant material used here consisted of a local sorghum grains called “Madjéri” from the group of local “Muskwari” sorghum grain (Folefack & Abou, 2016). The samples come from trial of the PROJET4 C2D‐SORGHO, from seeds harvested during 2015 crop year. This ecotype was chosen because of its good toughness (66 mm), its good extensibility (22 mm), combined with a poor baking strength (86.10–4 J) (Darman, 2013; Roger, Richard, & Francois‐Xavier, 2008). These characteristics are said to be suitable for biscuits production (Roger et al., 2008). Ingredients used to produce the biscuits included “Amigo” type 55 wheat flour, “Jadida” brand margarine, white powdered sugar, fresh chicken eggs, baking powder, and yeast.

### 2.2 Production and technological characterization of sorghum flour

**FIGURE 1:**
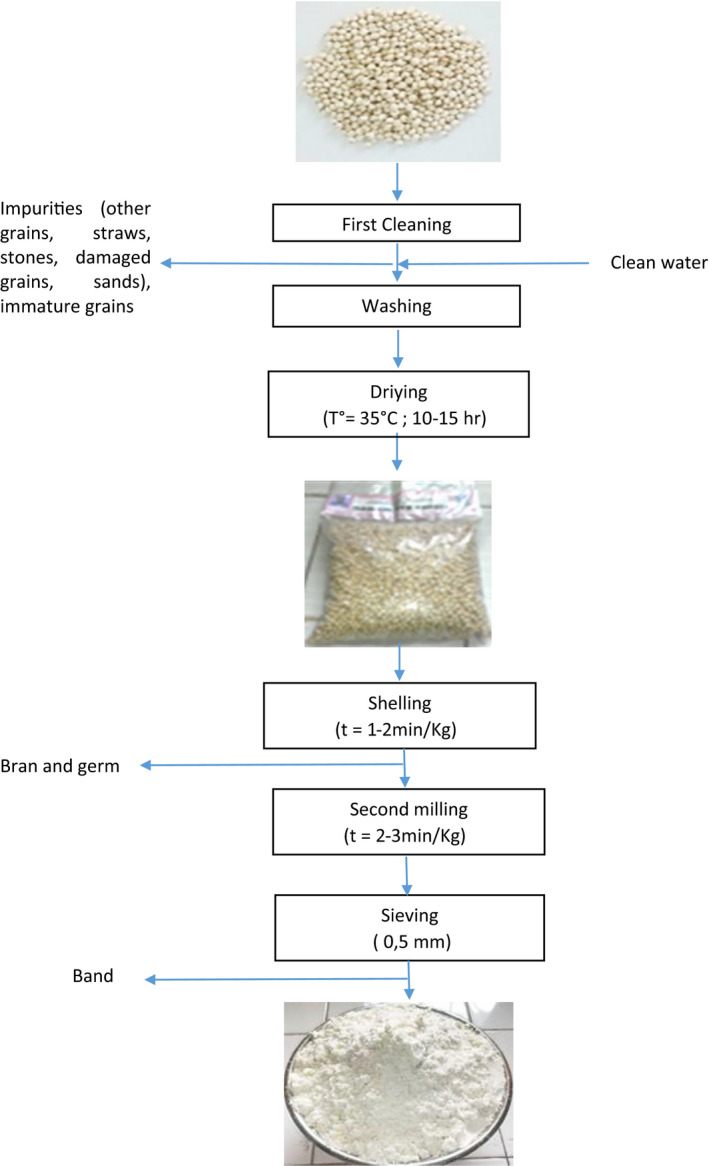
Process production of *Madjéri soghum* flour

After operations of cleaning and shelling, sorghum flour are produced by milling grains in an MGE3 hammer mill and the powder obtained is sieved through a sieve of 0.5 mm mesh (Figure 1). The technological characteristics of the sorghum flour obtained and the wheat flour purchased on the market were determined. The water content was determined by the AACC method (1995). The water absorption capacity, the solubility index, and the swelling rate were, respectively, determined by the method Philips, Chinnan, Branch, Miller, and Mc Watters (1988); the method of Anderson et al. (1969); the method of Okezie and Bello (1988) respectively.

### 2.3 Setting up of the technology production

The formulation used in the production of sorghum flour biscuits and or sorghum–wheat composite flour was as follows: 100 g of flour, 56.7 g of margarine, 56.7 g of sugar, 5.3 g of egg, and 0.5 g of baking powder.

The preparation consist at first weighting the ingredients as indicated before mixing them together for 2–4 min until a light dough is obtained. This last dough is shaped and baked at 200°C for 45 min.

Before the introduction of biscuits dough inside oven, the shaped dough are weighed and the specific volume is determined by the formula VspBF = *πe* (*D* ^2^)/4 where *e* is the thickness of the biscuit and *D* is its diameter (Edelstein & Schachman, 1973).

After beating and draining, the biscuits are weighed once more, the mass losses and the specific volume are determined.

### 2.4 Sensory analysis

A hedonic test was organized to evaluate the general appreciation of biscuits in real consumption conditions. Based on their interest in the study and their availability, 60 subjects aged between 15 and 25, consumers of shortbread biscuits were recruited for this sensory analysis. The hedonic scale used was that of nine points developed by Chandra, Singh, and Kumari (2015), ranging from huge like (9) to hate (1), to like (8), moderate like (7), like a little (6), indifferent (5), does not like much (4), does not like (3), does not like at all (2). The criteria for this assessment were taste, texture, color, smell, and overall appreciation.

### 2.5 Nutrition labeling

The approximate nutritional composition of biscuits was determined using the AlimenthèqueTM 5.0 nutritional analysis and calculation software (Nunes, Alvarenga, Souza Sant'Ana, Santos, & Granato, 2015). The quantities of ingredients and the recipe are introduced into the software, and the nutritional and energy inputs are generated.

### 2.6 Statistical analysis

All means were calculated by excel software, and R‐software was use to proceed statistical analysis (Chambers, 2008).

## 3 RESULTS AND DISCUSSION

### 3.1 Sorghum flour production

Flour production was carried out according to the steps and parameters described in Figure 1. Given the equipment used (mill and hammer mill), the dehulling and milling operations were carried out in duplicate. The hulling was done in a hulling machine with an hourly output of 1–2 min/kg of grain, the huller being coupled with a separator to separate the hulled grains, bran, and eliminated germ. The milling was done in duplicate in a hammer mill. Grinding grain to a finest grain, this is in order to better improve the hydration properties of the sorghum flour obtained.

After the grinding the grains, the product obtained is sieved and the produced flour with particles sieve of mesh 0.5 mm (NC 88 : 2002‐03; CODEX STAN 172‐1989, 173‐1989 Rev^1‐1995^) is obtained. This mesh was chosen not only because it was prescribed for fine quality sorghum flour, but also in relation to a sandy texture needed for biscuits. The sieving operation which aims at separating the useful fraction is carefully carried out using stainless steel laboratory sieves, in accordance with good hygiene practices (wearing a gown, an oral mask, cleaning, and disinfection of equipment). We obtained a whitish powder consisting of particles with a size of 0.5 mm and less (residual flour) on which depends the particle size and the texture of the final biscuits.

### 3.2 Technological characterization of used flours

The technological characterization concerned the sorghum flour produced by us and the type 55 wheat flour purchased on the market. Analysis were conducted in triplicate for each parameters namely: water content (TE), water absorption capacity (CAE), solubility index (SI), and swelling rate (TG). The means and standard deviations of the results obtained for each parameter and for each flour are recorded in Table 1:

**TABLE 1:** Technological characteristics of produce flours

Item (%)	Wheat flour	Sorghum flour
TE	11.27 ± 2.6	8.33 ± 2.36
CAE	148.15 ± 6.4	102.26 ± 6.41
IS	16.06 ± 5.3	20 ± 5.25
TG	101.92 ± 4.25	102.94 ± 5.2

The difference recorded between the water absorption capacities of these two flours is probably related to the physicochemical characteristics of its constituents. Indeed, the water absorption capacities is an essential technological parameter to control the consistency of the dough (Bchir, Rabetafika, Paquot, & Blecker, 2014; Villemejane, Roussel, Berland, Aymard, & Michon, 2013). It reflects the hydration capacity of flour in the presence of liquid water and depends mainly on moisture, particle size, starch damage rate, and protein content (Barros, Telis, Taboga, & Franco, 2018; Berton, Scher, Villieras, & Hardy, 2002). We can guess that wheat flour contains more damaged starch than sorghum. Both the solubility index and the water absorption capacities predict the behavior of a flour when it is kneaded with water at room temperature, except that it refers much more to the solubility of the flour proteins (Franco‐Miranda, Chel‐Guerrero, Gallegos‐Tintoré, Castellanos‐Ruelas, & Betancur‐Ancona, 2017; Vogel, Scherf, & Koehler, 2019). The more a flour has a high solubility index, the more it mixes with water in the liquid phase and at room temperature. The superiority of the solubility index of sorghum flour over that of wheat can be explained by the presence in wheat flour of less free sugars and gluten which is a protein derived from mixture of glutelins and gliadins two insoluble proteins in water (Jribi, Sahagùn, Debbabi, & Gomez, 2019). The rate of swelling refers to the ability of a flour to grow when sufficiently hydrated. The superiority of the value of this parameter in sorghum flour over that of wheat could be explained by the high content of sorghum flour in nonstarchy carbohydrates (Chavan et al., 2017) such as pentosans. Indeed, it is recognized that pentosans would absorb between 5 and 15 times their weight in “liquid” water (Apollonia, 1971). Their mechanism of action is to absorb water as dietary fiber, to swell (Avramenko, Tyler, Scanlon, Hucl, & Nickerson, 2018; Cauvain, 2015). This swelling is therefore largely responsible for the high swelling of sorghum flour relatively to that of wheat. A biscuit whose dough contains more sorghum flour than that of wheat may be better ventilated and less dense than a biscuit richer in wheat flour, and therefore will have a better texture.

### 3.3 Processing of shortbread biscuits made from sorghum flour

**FIGURE 2:**
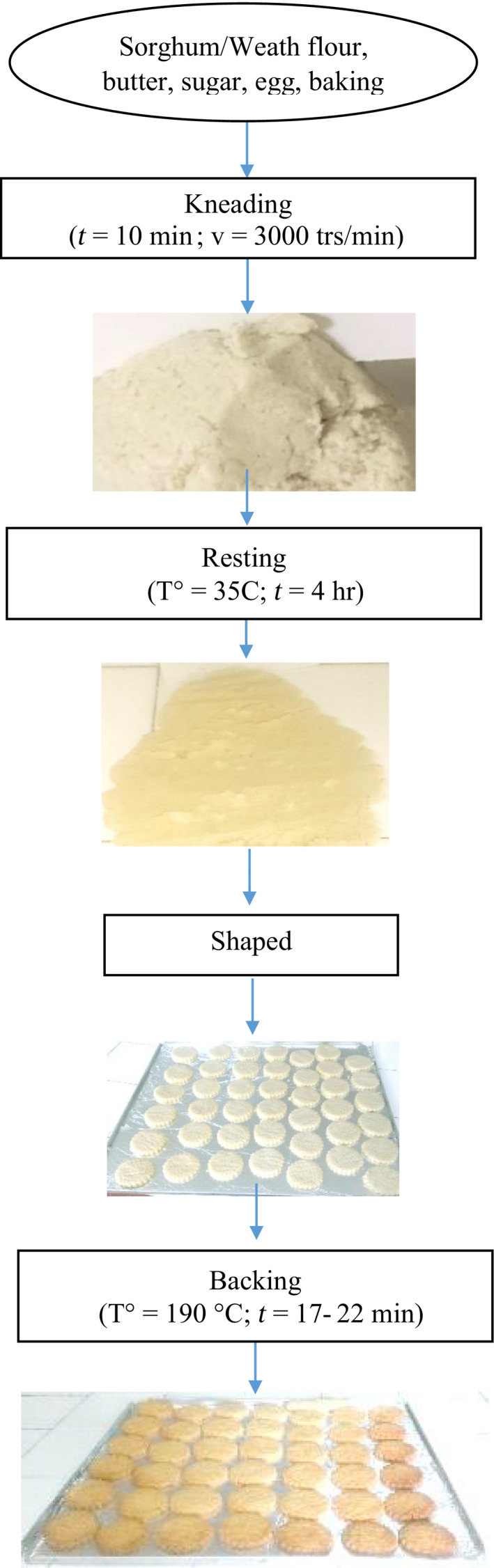
Processing of shortbread biscuits made from sorghum flour

The process shown in Figure 2 is the one adopted for the production of the biscuits at 100% sorghum flour. Considering the poor hydration properties of sorghum flour, the kneading time was increased to 10 min and the kneading speed was increased to 3,000 rpm. Indeed, kneading forces water to first wrap each particle of flour, then to enter (Adedeji, Joseph, Plattner, & Alavi, 2017; Wang, Ai, Hood‐Niefer, & Nickerson, 2019). The faster the kneading, the more important the phenomenon (Othman, 2007). Thus, the more we increase the kneading speed, better the flour particles are hydrated and less the biscuits obtained after cooking may include raw starch granules. The mixing time adopted is that at the end of which the mixture was homogeneous, and above which the resulting sandy paste began to lose its consistency and become more and more flaccid and sticky. However, it was impossible to obtain a paste of desired consistency at the end of kneading. The mixture obtained was very flaccid, dense, adhering to the hands and walls of the kneader and impossible to work. It had the consistency of a pasta dough and not that of a biscuit dough.

To overcome this problem, a step of steeping the dough was added to the process. Due to the poor water absorption capacities of sorghum flour, the granules of its starch will take longer to absorb water. This time is given to them during this stage which may allow completion of the water absorption process started during kneading. Thus, the consistency required for a biscuit dough must be obtained at the end of this step, in order to allow shaping of dough. The length of the resting period of dough retained for the production of these 100% sorghum biscuits was four hours because after this time interval, the consistency of dough no longer changed. Even at the end of this resting period, the consistency indicated for biscuit dough was not reached and the dough remained impossible to work (Figure S1).

At the end of this production of biscuits at 100% sorghum flour, the biscuits obtained were not satisfactory from the point of view of the quality criteria for a biscuit (Stefanova, 2017). They had a very dense texture, crumbled to touch and showed cracks on their upper surface, hardened immensely immediately after cooling (Figure S2).

In addition to the poor hydration properties of sorghum flour, the following reasons have been cited to justify the poor quality of these biscuits:

- The absence of gluten in sorghum flour: In biscuits, lightness, friability, taste, and texture of the products are mainly due to gluten, which is the main protein characterizing wheat flour that is usually used in this field (Benkadri, 2010). In fact, during the kneading and baking of the biscuits, the proteins of the gluten pool and create around the dough a viscoelastic network responsible for retaining the gas evolved either by the fermentative activity of the yeasts, or by the action of the leavening powders, under the action of heat (Hackenberg, Vogel, Scherf, Jekle, & Becker, 2019; Öhgren, Fabregat, & Langton, 2016). The more the flour contains this protein, the denser the viscoelastic network around the dough, the better the texture of the products.
- The rapid degradation of starch: During gelatinization, if the overall water content is limited (<30%) as in the production of these 100% sorghum flour biscuits, then the distribution of water within the grain is uneven (poor starch hydration capacity). Crystallites near water‐rich amorphous zones would gelatinize while others would melt at higher temperatures, until only a single‐grain melting occurred (Jenkins & Donald, 1998; Rahman & Roos, 2017). The studies conducted by Balla, Blecker, Oumarou, Paquot, and Deroanne (1999) on the aging profile of breads made from sorghum and wheat composite flour showed that for strong intakes (>80%), the staling of bread evolves very rapidly. This rapid evolution maybe link to the structure of the crumb which in turn is influenced by the degree of gelatinization of the starch (Martínez, Román, & Gómez, 2018). Since sorghum has a low gelatinization, because poorly hydrated, breads incorporating a large amount of sorghum harden faster because rapid retrogression of its amylose. Thus, its poor hydration during kneading, mainly because of insufficient water (Balla et al., 1999) leads to partial gelatinization and therefore a rapid retrogression, once at room temperature.

The production of 100% sorghum biscuits of acceptable quality is not possible. In order to produce biscuits of satisfactory quality, we proposed to produce biscuits based on composite flour resulting from the substitution of an amount of wheat by sorghum flour, in the formulation of biscuits. In the formulation of biscuit products, gluten is an essential structuring protein, contributing to the appearance and structure of products (Benkadri, 2010), which makes the technological feasibility of gluten‐free products particularly difficult. The step chosen between two consecutive substitution rates was ten because it is the one from which a more or less observable difference in the consistency and color of the dough was noted as mentioned in the work of Akintayo and Sedgo (2001), Farzana and Mohajan (2015), IFAD (2001), Ali and Djalé (2001), Ouattara et al. (2001), Ndoye (2001) during similar work on the production of bread or biscuits based on sorghum/wheat composite flour. By varying parameters such as time and speed of kneading, the resting times of the dough, biscuits ranging from 10% to 90% substitution and the control biscuit (100% wheat flour) were produced. It has been found that the production parameters vary with the substitution rate (Table 2).

**TABLE 2:** Average dough and dough rest times based on substitution rate

Substitution rates (%)	Duration of kneading (min)	Resting time of the dough (min)
10	10 ± 1.8^a^	300 ± 2^a^
20	9 ± 1.3^a^	210 ± 1^b^
30	7 ± 1.5^ab^	180 ± 1.2^b^
40	7 ± 0.8^b^	22 ± 0^c^
50	5 ± 0.6^c^	19 ± 0.6^c^
60	4 ± 1^dc^	17 ± 1^cd^
70	4^d^	16 ± 0.6^d^
80	4 ± 0.5^d^	13 ± 0.6^de^
90	3 ± 1^d^	11 ± 1^e^
100	3 ± 1^d^	0^f^

Same lower‐case letters in superscript indicate no statistical significance diference between means.

The decrease in the kneading time here reflects the increase in water absorption and the stability of the dough with the substitution. This could be explained by the low protein content of sorghum flour and by a probable low level of damage sorghum flour (Balla et al., 1999). It is also noted that the decrease of the kneading time is slowed down considerably when exceeds 50% of substitution that is to say when wheat flour is more than sorghum flour in the mixture. This may be explained by the fact that wheat flour has better hydration properties than sorghum flour (Alavi et al., 2019). The negative evolution relation of the resting time of the pulp and the rate of substitution could be explained by the poor hydration properties of sorghum flour compared with that of wheat, bad properties whose reasons were mentioned above.

#### 3.3.1 Sensory quality

**FIGURE 3:**
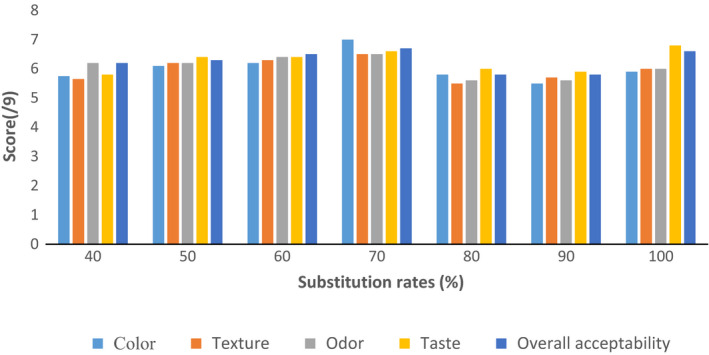
Hedonic biscuits notation based on substitution rates

Biscuit control and those of substitution rates ranging from 40% to 90% were submitted to the panelists' appreciation. The results indicate that all biscuits presented to panelists were accepted, with all grades averaging over 6, on an acceptance scale ranging from 0 to 9. Biscuits preference was for the biscuits made from flour made up of 70% wheat flour. This result is similar to that observed by IFAD (2001), in a similar study of the N'Ténimissa variety of sorghum from Mali (Akintayo & Sedgo, 2001). Biscuits obtained at substitution rates ranging from 40% to 60% were found to be of acceptable quality up to 96.67% for 100%; 90%, and 50%; 93.33% for the 60% and 40% rates; 86.67%; 98.33%, respectively, for the rates of 80% and 70%. The hedonic notation of biscuit by the panelists gave the results shown in Figure 3.

**FIGURE 4:**
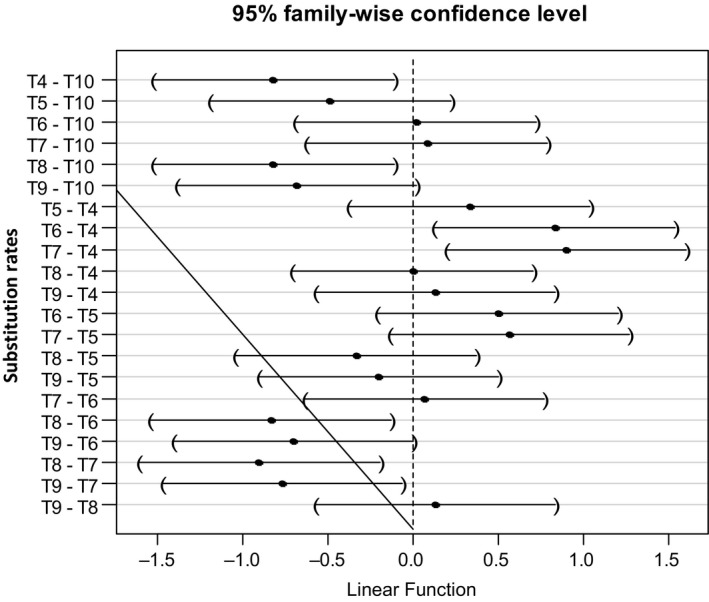
Analysis of variances of Hedonic biscuits scores according to the substitution rates. T*x* = *x*.10%. NB: Two means are significantly different if 0 does not belong to the confidence interval of their difference (*p* < .05)

Biscuits with 70% wheat flour incorporation had the highest average scores for the overall preference criteria (6.97 ± 1.30), color (7.1 ± 1.45), and texture (6.62 ± 1.54). For the odor and taste criteria, the 60% biscuits and the control received the highest average scores, namely 6.77 ± 1.55 for odor and 7.12 ± 1.29 for taste. Similar results were observed during the work of Akintayo and Sedgo (2001) and IFAD (2001). A significant influence of the substitution rate on the sensory appreciation of biscuits was observed at (*p* < .001). At the significance level of 5%, the analysis of variances in multiple comparison of the scores assigned to the different substitution rates allowed us to obtain the results presented in Figure 4.

This analysis of the variances shows us that, apart from T7 and T8, a significant difference is observed only between two substitution rates separated by at least two substitution steps (T4 and T10, T8 and T10, T6 and T4, T7 and T4, T8 and T6, T9 and T7, and T9 and T6). This insignificant difference between two consecutive substitution rates may be due here to the fact that sorghum and wheat flours, like the flours of most cereals, do not have “of taste” characteristics. It could indicate a good choice made at the level of substitution, good conduct of the operations of production of biscuits and the level of sensory tests, the bias from the tasters.

#### 3.3.2 Nutrition labeling

The proximate comparative nutritional composition of the 100% wheat flour biscuit and the one most appreciated substituted biscuit by panelists (70% substitution) is given in Table 3.

**TABLE 3:** Proximate composition and energy intakes of *muskari* biscuits

Macro nutriments (g/100 g)	100	70
Proteins	5.65 ± 1.00	5.22 ± 0.32
Lipids	21.72 ± 2.23	22.7 ± 2.32
Sugar	57.50 ± 1.05	58.51 ± 1.02
Saturated	4.30 ± 1.06	4.35 ± 0.95
Sodium	0.067 ± 0.00	0.067 ± 0.00
Fibers	1.78 ± 0.01	2.15 ± 0.00
Energy (kcal)	451.4 ± 12.56	454.1 ± 11.23

Similar energy intakes (421 kcal) were observed for the 20% sorghum biscuits produced by Akintayo and Sedgo (2001), during their work on the production of N'Ténimissa a Malian sorghum biscuits. It should be noted here that for the same basic composition, the biscuit containing 30% of sorghum flour may provide its consumer with more energy than the control biscuit. This difference in nutritional composition can be explained mainly by the difference in biochemical composition between sorghum and wheat flour. Indeed, for the same quantity of flour, that of sorghum would provide 76.64% of digested carbohydrates and 3.34% of lipids against 69.3% of digested carbohydrates and 0.8% of lipids for wheat flour (Alimentheque, 2016). The high dietary fiber intake of sorghum biscuits compare to wheat is probably related to the high fiber content of sorghum flour (6.6%) compared with wheat (3.9%) (Alimentheque, 2016; Stadlmayr, 2012).

## 4 CONCLUSION

This work aims at valorizing a local sorghum produced in Cameroon by producing biscuits. Sorghum flour was produced from *Madjéri* sorghum grains. The technological characterization of this flour allowed us to notice that it had poor hydration properties, with a water absorption capacity of 102.26 ± 6.41% and a solubility index of 20 ± 5, 25%. The 100% sorghum flour biscuits produced by changing the kneading time and incorporating the resting time of the dough in the process of making shortbread type biscuits were considered to be of poor technological quality. Because of its high gluten content, the partial substitution of sorghum flour with that of wheat in the biscuit formulation may improve technological properties such as lightness and friability. Up to 30% substitution, biscuits remain of poor technological quality.

Panelists preferred the 70% substituted sorghum flour biscuit. It also appears from this study that the biscuit at 70% sorghum flour preferred by the panelists is more energetic than biscuit at 100% wheat flour. This can lead to a light and rich biscuits that can add value to local grains while meeting new needs for light and rich food in urban areas.

## Supporting information

Figure S1‐S2
