# Supplementary material for: Development of sorghum‐based shortbread biscuits from “muskwari” flour
Source: Food Sci Nutr. 2020 May 29;8(7):3181–9. doi: 10.1002/fsn3.1574 (PMC7382165; doi:10.1002/fsn3.1574)
Supplement: Supplementary file 1 — Figure S1‐S2 [file FSN3-8-3181-s001.docx]

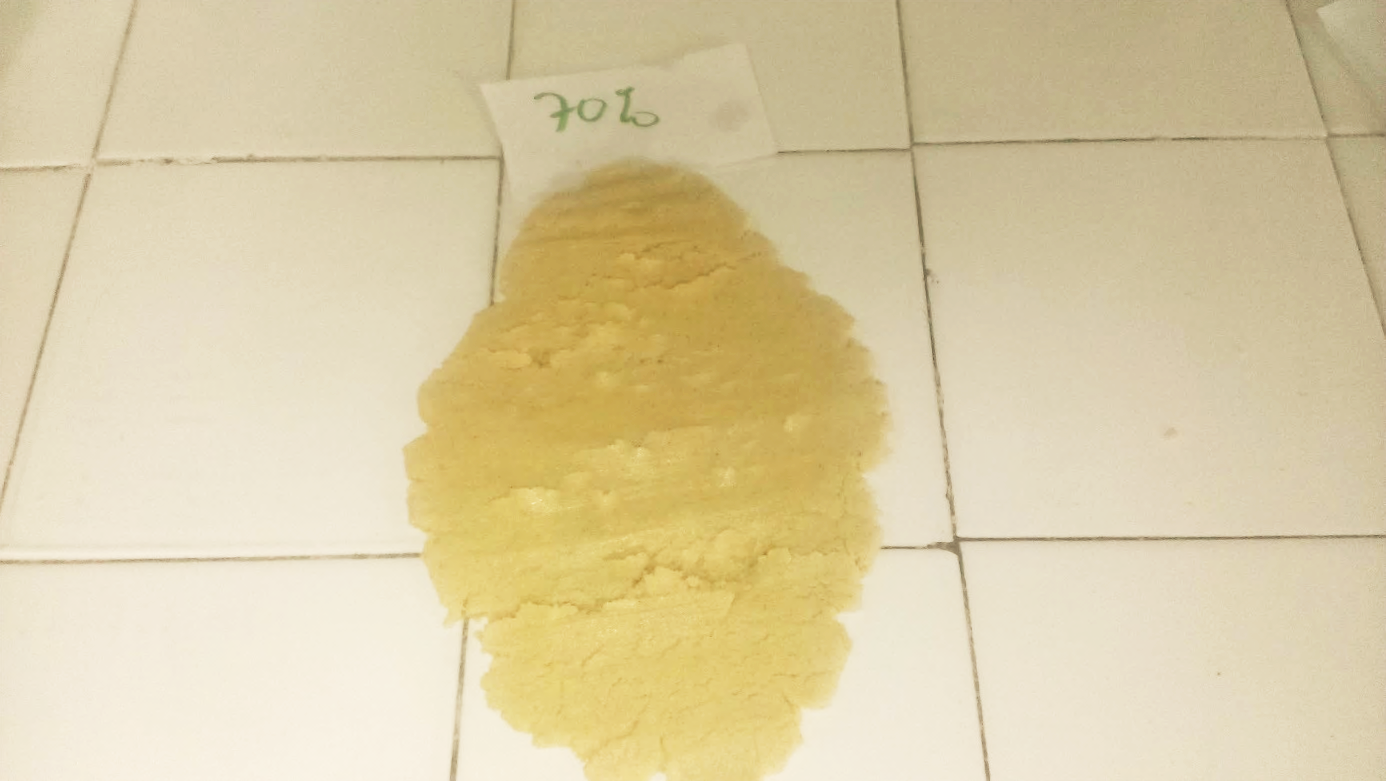


**Figure S1 :** Cooked dough of 100% sorghum flour biscuits

**A**

**B**

**Figure S2 :** Biscuits 100% sorghum flour with a cracked upper surface (A) and a high density (B)
